# Epidemiology of carbapenem resistance among multi-drug resistant Enterobacteriaea

**DOI:** 10.1186/2047-2994-4-S1-P133

**Published:** 2015-06-16

**Authors:** V Katawera, L Ampaire

**Affiliations:** 1 Microbiology, Mbarara University, Mbarara, Uganda; 2 Laboratory, Epicentre Mbarara Research Centre, Mbarara, Uganda; 3 Medical Laboratory Sciences, Mbarara University, Mbarara, Uganda

## Introduction

Multi-drug resistant (MDR) *Enterobacteriaceae* are on the increase worldwide and their spread has become a global challenge. Escalating the challenge is the possibility that many of these are Carbapenemase-producing *Enterobacteriaceae* (CPE). This further complicates patient management. The magnitude of MDR-CPE in many developed settings has been reported, however, there is paucity of data from resource limited settings.

## Objectives

We evaluated the epidemiology of MDR-CPE of clinical origin in South Western Uganda.

## Methods

From September 2013 to June 2014, all *Enterobacteriaceae* isolated from diverse specimens obtained from patients attending Mbarara Regional Referral Hospital, South-western Uganda, were screened for MDR in a laboratory-based cross sectional study. Isolates found to be MDR were screened for carbapenem susceptibility/resistance phenotypically by Kirby Bauer disc diffusion method following EUCAST guidelines and genetically using the multiplex real-time Polymerase Chain Reaction (RT-PCR).

## Results

Of the 658 strains isolated, 183 (27.8%) were MDR and 68 (37.15%) of those MDR exhibited at least one form of carbapenem resistance with 23 (12.57%) and 56 (30.60%) isolates expressing phenotypic and genetic resistance, respectively. Eleven MDR-CPE (6.01%) isolates exhibited both phenotypic and genotypic resistance to carbapenems. Only *bla*VIM and *bla*OXA-48 genes were detected among the genetically resistant isolates.

## Conclusion

The high prevalence of MDR-CPE calls for aggressive infection control and prevention strategies, including reinforcement of hand hygiene, using contact precautions and early detection of CPE through use of targeted surveillance and molecular techniques in resource limited settings.

## Disclosure of interest

None declared.

